# Compensated Accidents

**Published:** 1913-03-22

**Authors:** 


### OUR NATIONAL INSURANCE ACT DEPARTMENT.

### XLVII.—Compensated Accidents.

It is to be feared that the provisions of the National Insurance Act relating to the payment of sickness benefit when the insured person is rendered incapable of work by an injury or disease which would entitle him to claim compensation or damages, whether from his employer or from any other person, are as yet but faintly realised either by employers or insured persons. Too wide a publicity cannot be given to the matter, as it is one that not only affects the operations of the approved societies but has an important bearing on the necessity for insurance against accidents under the Workmen's Compensation Act and under the Employers' Liability Act.

The provisions of the Act relating to this subject are contained in Section 11, the purport of which is that if an insured person is entitled to obtain compensation or damages for the injury or disease from which he is suffering, he can only claim payment from his approved society for such an amount as will make up the difference between the sickness benefit under the Act and the weekly value of the compensation to which he is entitled.

The reasons for this are twofold. In the first place the contributions payable under the Act were framed on the actuaries' estimates, which definitely excluded the accident risk from the sickness tables they employed. In the second place the existing legislation embodied in the Workmen's Compensation Act and the Employers' Liability Act had already placed the responsibility for accidents on the employers' shoulders, and it was not intended to relieve them of their liability by means of the National Insurance Act.

### A Position not yet Realised by Employers and Insured Persons.

We stated at the outset that it was to be feared that the provisions of the Act relating to this matter are as yet but faintly realised. This statement is based on the result of inquiry from one of the largest of the approved societies, from which we learn that approximately 10 per cent. of the claims received since the benefits became payable have shown that the incapacity for work has arisen by accident or injury for which compensation could be claimed. It is worthy of note in passing that the estimate placed by the actuaries on the accident risk included in the standard sickness tables was the same allowance of 10 per cent. Only very inadequate data was in existence to enable them to arrive at this allowance, and it may only be a coincidence that the claims so closely approximate to their estimate, but the fact is significant nevertheless. If the provisions of the Act had been realised it is probable that a large number of the claims on the approved society would never have been made. In order to cope with the matter it has been necessary to set up a special department, and it is interesting to note the methods adopted.

### The Attitude of the Approved Societies.

The necessity for the exercise of care by the approved societies in the matter is at once apparent; for, in the first place, it is clear that a society cannot afford to pay anything like 10 per cent. more in claims than those provided for in the contributions it receives; and, secondly, if it did actually pay it would stand the risk of having the payment disallowed by the auditors appointed by the Treasury. In this connection it should be pointed out that the provision of the Act is an explicit statement that "no sickness benefit or disablement benefit *shall* be paid" in the circumstances with which we are dealing, and no option is given to the society, though by sub-section (3) of the same section power is given to a society to make payments by way of advance pending the settlement of claim for compensation or damages, reserving the right to recovery of the advance when the settlement is made.

The method usually adopted when a claim for sickness benefit is put forward which appears to indicate to the officers of the society that the insured person is entitled to compensation, is to inform him of the fact, and to refer him to his employer or to the person from whom the damages are recoverable. This does not always immediately result in a settlement. In many cases it has been found that the employer has repudiated liability, and has referred the insured person back to his society. If this should occur it immediately becomes necessary for the full circumstances of the case to be gone into, and legal advice is given gratuitously to the member to enable him to support his claim for compensation. Experience has already shown that this advice has been appreciated, and, further, that the results achieved thereby have been satisfactory not only to the society but also to the members directly concerned, for in many cases the amount of compensation which can be claimed exceeds the sickness benefit payable under the Act.

It would be possible for the society in an extreme case to take action on behalf of a member against his employer when he refuses or neglects to take proceedings to enforce his claim. There are, however, certain obvious disadvantages in such a course; for, in the event of the failure of such proceedings, the costs incurred would have to be borne by the society as if it were claiming on its own account. Furthermore, the institution of legal proceedings would almost necessarily create strained relationship between the employer and the member, and the latter might conceivably find himself in a worse position by being thrown out of employment than if no action had been taken on his behalf.

It is easy to see, therefore, that matters of this description require handling with considerable tact and circumspection by the officers of the approved societies.

This is one of the advantages of membership in a large and well organised society that was generally unforeseen, but which may prove to be by no means the least of their attractions. These large societies have at their disposal the services of legal experts, and they are able to specialise in a way that is quite outside the bounds of possibility in the case of small societies.

### How the Question Concerns Employers.

We have said enough to show that the matter is receiving the careful consideration of society officials, but it will be perceived that the question has further significance as far as employers of labour are concerned. Most employers of labour on a considerable scale have already realised the liability imposed on them by the Employers' Liability Act and similar enactments, and have provided against that liability by means of insurance. Employers of labour on a more moderate scale have not to the same extent availed themselves of insurance protection, and in particular, though a big sensation was aroused in 1906 by the inclusion of domestic servants in the scope of the Workmen's Compensation Act, and the insurance offices had almost more than they could do at the time to cope with the business that came to them, it is common knowledge that a large proportion of the employers of domestic labour are serenely oblivious to the risks they run by neglecting to insure their servants against accident. It has to be recognised that in the past many accidents have occurred which would undoubtedly have entitled the servant or employee to claim compensation, but through lack of knowledge such claims have not been preferred, and the employers have not suffered by their neglect of insurance provision. The provisions of the National Insurance Act with which we have been dealing will very soon put a different complexion on the matter, for, as we have pointed out, the approved societies must, for their own protection, acquaint their members with their rights against their employers, and the latter will be forced in self-defence to resort to the protection of an accident insurance policy.

This aspect of the question has already found expression in the reports of accident insurance companies, and these business enterprises will not be slow to take full advantage of the accession of new and profitable business.

### Medical Benefit for Nurses.

In our issue of March 1 we dealt at length with the attitude of Insurance Committees towards medical benefit, and were able to make the gratifying announcement that the claims of hospital nurses to receive special consideration in regard to making their own arrangements with the resident medical officer of the hospital, instead of having to place their names on the list of panel doctors, had at last been recognised. This was signalised, as we pointed out, by the issue of sheaves of forms and instructions by the Insurance Commissioners, some of which we stated were so complicated that they would become dead letters and useful only as an excuse to the Insurance Committees for doing nothing. It is to be feared that our forecast has proved only too true, for we learn from an esteemed correspondent that an application to the Derbyshire Insurance Committee on behalf of the nurses engaged in a hospital within their area was declined in the following terms: "Each County Committee is authorised to decide whether it will permit independent arrangements to be made by any insured persons in its area with doctors not on the panel. My Committee have decided that no such independent arrangements can be permitted in the County of Derby. It will, therefore, be necessary for all insured persons within this area to have their names placed on the list of a panel doctor."

Thus, it would seem that each Insurance Committee is to be a law unto itself, and we cannot refrain from expressing our opinion that this is most unsatisfactory. There does not seem to us to be any valid reason why any Insurance Committee should decline to follow the reasonable lead given in the matter by the London Insurance Committee.

In order to obviate any possible misconception it should be pointed out that permission to make their own arrangements must be obtained by the nurses, or by the hospital authorities on their behalf, from the local Insurance Committee, and it is desirable that applications should be made at once if they have not already been forwarded.

